# Supplementary material for: A deep learning method for simultaneous denoising and missing wedge reconstruction in cryogenic electron tomography
Source: Nat Commun. 2024 Sep 23;15:8255. doi: 10.1038/s41467-024-51438-y (PMC11420219; doi:10.1038/s41467-024-51438-y)
Supplement: Supplementary file 1 — Supplementary Information [file 41467_2024_51438_MOESM1_ESM.pdf]

Supplementary Information

1 Background on Self-Supervised Deep Learning Techniques

The DeepDeWedge loss is inspired by Noise2Noise [1] and Noisier2Noise [2] self-supervised learning. Here, we give a brief overview of the main ideas behind these two frameworks.

Denoising with Noise2Noise

Noise2Noise is a framework for constructing a loss function that enables training a neural network for image denoising without ground-truth images. Neural networks for denoising are typically trained in a supervised fashion to map a noisy image to a clean one and thus require pairs of clean images and corresponding measurements.

Noise2Noise-based methods also aim to train a neural network to map a noisy image to a clean one. Contrary to supervised learning, Noise2Noise assumes access to a dataset of pairs of noisy observations $\mathbf{y}_i^0 = \mathbf{x}_i^* + \mathbf{n}_i^0$ and $\mathbf{y}_i^1 = \mathbf{x}_i^* + \mathbf{n}_i^1$ for each ground-truth image $\mathbf{x}_i^*$. Moreover, it assumes that the noise terms $\mathbf{n}_i^0$ and $\mathbf{n}_i^1$ are independent and that the noise $\mathbf{n}_i^1$ is zero-mean. Then, one can train a network $\mathrm{f}_{\boldsymbol{\theta}}$ for denoising to map the noisy observation $\mathbf{y}^0$ onto its counterpart $\mathbf{y}^1$. Formally, one can show that

$$\mathop{\mathbb{E}}_{\mathbf{y}^0,\mathbf{y}^1}\left[\left|\left|\mathrm{f}_{\boldsymbol{\theta}}(\mathbf{y}^0) - \mathbf{y}^1\right|\right|_2^2\right] = \mathop{\mathbb{E}}_{\mathbf{y}^0,\mathbf{x}^*}\left[\left|\left|\mathrm{f}_{\boldsymbol{\theta}}(\mathbf{y}^0) - \mathbf{x}^*\right|\right|_2^2\right] + c, \tag{1}$$

where c is a constant that is independent of the network weights $\boldsymbol{\theta}$. Thus, a self-supervised Noise2Noise training objective based on samples approximates, up to an additive constant, the same underlying risk (left side of Equation (1)) as the supervised objective function. This approximation becomes better as the number of training examples increases and theoretically and empirically, networks trained with a Noise2Noise-like loss perform as well as if trained on sufficiently many examples [3].

Denoising and Recovering Missing Data with Noiser2Noise

IsoNet's denoising approach is motivated by the Noisier2Noise framework [2]. While training a model for image denoising with a Noise2Noise objective requires paired noisy observations, a Noisier2Noise objective is based on a single noisy observation per image.

Noisier2Noise for Denoising: We assume that we have access to a dataset of noisy images. Contrary to Noise2Noise, we assume that we have only one single noisy observation $\mathbf{y} = \mathbf{x}^* + \mathbf{n}$ per ground-truth $\mathbf{x}^*$. The goal is to train a neural network for image denoising. For constructing a training objective with Noisier2Noise, we assume that the noise terms $\mathbf{n}$ that corrupt all images come from the same distribution and that we are able to sample from this distribution. We construct model inputs $\tilde{\mathbf{y}}$ by further corrupting the noisy images $\mathbf{y}$ with an additional noise term $\tilde{\mathbf{n}}$ sampled from the true noise distribution, i.e., $\tilde{\mathbf{y}} = \mathbf{y} + \tilde{\mathbf{n}}$. Moran et al. [2] showed that if we train a neural network $\mathrm{f}_{\boldsymbol{\theta}}$ to map these noisier model inputs $\tilde{\mathbf{y}}$ onto their single noisy counterparts $\mathbf{y}$ using the squared L2-loss, one can use the final trained network to construct a denoiser for the double noisy images $\tilde{\mathbf{y}}$.

Noisier2Noise for Missing Data Recovery: We use ideas from Noisier2Noise for missing wedge reconstruction. To illustrate how Noisier2Noise can be used to predict missing data, consider the following setup: Assume we have a dataset of masked measurements $\mathbf{y} = \mathbf{M}\mathbf{x}^*$. Here, $\mathbf{M}$ denotes a random mask, i.e., a diagonal matrix with entries in $\{0, 1\}$, and $\mathbf{x}^*$ is an unknown ground-truth signal vector. Like above, the ground truths and masks are different for each measurement $\mathbf{y}$ and the dataset contains only the measurements and no ground truths. In this setup, we want to train a neural network $\mathrm{f}_{\boldsymbol{\theta}}$ to predict the data $\mathbf{x}^*$ from the measurement $\mathbf{y}$. To do this, we construct double-masked measurements $\tilde{\mathbf{y}} = \tilde{\mathbf{M}}\mathbf{y}$, where $\tilde{\mathbf{M}}$ is another random mask, and train the network to map the double-masked measurements $\tilde{\mathbf{y}}$ to their counterparts $\mathbf{y}$. It can be shown that, under certain assumptions on the random masks $\mathbf{M}$ and $\tilde{\mathbf{M}}$, the final trained model can be used in an algorithm to recover the data missing in the singly masked measurements $\mathbf{y}$. As for denoising, the model estimates the missing data based on the doubly-masked measurement $\tilde{\mathbf{y}}$. For details, we refer readers to recent works by Millard and Chiew [4, 5], who discussed Noisier2Noise for the recovery of missing data in accelerated magnetic resonance imaging.

2 Normalizing Tomograms and Sub-Tomograms

During model fitting, we apply Gaussian normalization to each model input sub-tomogram $\tilde{\mathbf{v}}^0_{i,\boldsymbol{\varphi}_i}$ by subtracting a global mean μ and dividing by a global standard deviation σ, i.e.,

$$\tilde{\mathbf{v}}^0_{i,\boldsymbol{\varphi}_i} \leftarrow \frac{\tilde{\mathbf{v}}^0_{i,\boldsymbol{\varphi}_i} - \mu}{\sigma}. \tag{2}$$

We calculate the global mean μ and the global variance σ^2 from a set of M randomly sampled model inputs as

$$\mu = \frac{1}{M}\sum_{i=1}^{M} \text{mean}\left(\tilde{\mathbf{v}}^0_{i,\boldsymbol{\varphi}_i}\right), \qquad \sigma^2 = \frac{1}{M}\sum_{i=1}^{M} \text{var}\left(\tilde{\mathbf{v}}^0_{i,\boldsymbol{\varphi}_i}\right). \tag{3}$$

In addition to this sub-tomogram level normalization, we found it beneficial for the performance and stability of DeepDeWedge to also normalize the full tomograms during the final refinement step. We now discuss our normalization approach, which is relevant mainly for the case when applying DeepDeWedge to multiple tilt series.

Assume we want to obtain the DeepDeWedge reconstruction of a tilt series $\mathbf{t}$. For this, we have to apply a fitted model to sub-tomograms of the FBP reconstructions $\text{FBP}(\mathbf{t}^0)$ and $\text{FBP}(\mathbf{t}^1)$, where $\mathbf{t}^0$ and $\mathbf{t}^1$ are the result of splitting the tilt series $\mathbf{t}$ using the even/odd or frame-based split. Before extracting sub-tomograms from the reconstructions $\text{FBP}(\mathbf{t}^0)$ and $\text{FBP}(\mathbf{t}^1)$, we normalize them to have a mean $\mu_{\mathbf{t}}$ and variance $\sigma^2_{\mathbf{t}}$ via

$$\overline{\text{FBP}(\mathbf{t}^0)} = \frac{\text{FBP}(\mathbf{t}^0) - \text{mean}\big(\text{FBP}(\mathbf{t}^0)\big)}{\sqrt{\text{var}\big(\text{FBP}(\mathbf{t}^0)\big)}} \cdot \sigma_{\mathbf{t}} + \mu_{\mathbf{t}}, \tag{4}$$

$$\overline{\text{FBP}(\mathbf{t}^1)} = \frac{\text{FBP}(\mathbf{t}^1) - \text{mean}\big(\text{FBP}(\mathbf{t}^1)\big)}{\sqrt{\text{var}\big(\text{FBP}(\mathbf{t}^1)\big)}} \cdot \sigma_{\mathbf{t}} + \mu_{\mathbf{t}}. \tag{5}$$

For refinement, we extract sub-tomograms from these normalized reconstructions and then normalize the sub-tomograms with the global mean μ and the global variance σ^2 as in Equation (2). We calculate the normalization constants $\mu_{\mathbf{t}}$ and $\sigma_{\mathbf{t}}$ as in Equation (3), with the difference being that the sub-tomograms $\{\mathbf{v}^0_i\}_{i=1}^M$ that underly the sub-tomograms $\{\tilde{\mathbf{v}}^0_{i,\boldsymbol{\varphi}_i}\}_{i=1}^M$ are extracted exclusively from the FBP reconstruction $\text{FBP}(\mathbf{t}^0)$ of one half of the tilt series of interest $\mathbf{t}$. This ensures that the voxels of the normalized reconstructions $\text{FBP}(\mathbf{t}^0)$ and $\text{FBP}(\mathbf{t}^1)$ lie in a range similar to that of the model inputs during model fitting, which are generated from the reconstruction $\text{FBP}(\mathbf{t}^0)$.

To achieve this, one could alternatively set $\mu_{\mathbf{t}} = \mu$ and $\sigma^2_{\mathbf{t}} = \sigma^2$, where the mean μ and the variance σ^2 are the global ones used during model fitting, which were calculated from a sample of all sub-tomograms used for model fitting. However, our approach described above gives better results if the mean and variance of the tilt series $\mathbf{t}$ of interest are substantially different from the global mean μ and the global variance σ^2.

3 Proof of Proposition 1

We divide the proof of Proposition 1 into two steps. The first step, which we present as a lemma, is a result inspired by Noise2Noise and deals with the additive noise $\mathbf{n}^1$ on the model target. To facilitate notation, we assume from now on that all 3D objects are represented as column vectors in $\mathbb{R}^n$ for $n = N^3$, and that the masks $\mathbf{M}$ and $\tilde{\mathbf{M}}$ are random diagonal matrices with entries in $\{0, 1\}$.

Lemma 1. Assume that the noise term $\mathbf{n}^1$ is zero-mean and independent of the noise term $\mathbf{n}^0$ and the masks $\mathbf{M}$ and $\tilde{\mathbf{M}}$. Then

$$\mathop{\mathbb{E}}_{\mathbf{n}^1}\left[\left|\left|(\tilde{\mathbf{M}}\mathbf{M} + 2\tilde{\mathbf{M}}^C\mathbf{M})\mathbf{F}\Big(\mathrm{f}_\theta(\tilde{\mathbf{v}}^0) - \mathbf{v}^1_{xr}\Big)\right|\right|_2^2\right] = \left|\left|(\tilde{\mathbf{M}}\mathbf{M} + 2\tilde{\mathbf{M}}^C\mathbf{M})\mathbf{F}\Big(\mathrm{f}_\theta(\tilde{\mathbf{v}}^0) - \mathbf{v}^*\Big)\right|\right|_2^2 + c,$$

where $c > 0$ is a constant that does not depend on the weights $\boldsymbol{\theta}$.

Proof. [Proof of Lemma 1] We calculate

$$\begin{aligned}
\left|\left|\tilde{\mathbf{M}}\mathbf{M}\mathbf{F}\big(\mathrm{f}_\theta(\tilde{\mathbf{v}}^0) - \mathbf{v}^1_{xr}\big)\right|\right|_2^2 &= \left|\left|\tilde{\mathbf{M}}\mathbf{M}\mathbf{F}\big(\mathrm{f}_\theta(\tilde{\mathbf{v}}^0) - \mathbf{F}^{-1}\mathbf{M}\mathbf{F}(\mathbf{v}^* + \mathbf{n}^1)\big)\right|\right|_2^2 \\
&= \left|\left|\tilde{\mathbf{M}}\mathbf{M}\mathbf{F}\big(\mathrm{f}_\theta(\tilde{\mathbf{v}}^0) - (\mathbf{v}^* + \mathbf{n}^1)\big)\right|\right|_2^2, \\
&= \left|\left|\tilde{\mathbf{M}}\mathbf{M}\mathbf{F}\big(\mathrm{f}_\theta(\tilde{\mathbf{v}}^0) - \mathbf{v}^*\big)\right|\right|_2^2 \\
&\quad - 2\Big\langle\tilde{\mathbf{M}}\mathbf{M}\mathbf{F}\big(\mathrm{f}_\theta(\tilde{\mathbf{v}}^0) - \mathbf{v}^*\big), \tilde{\mathbf{M}}\mathbf{M}\mathbf{F}\mathbf{n}^1\Big\rangle \\
&\quad + \left|\left|\tilde{\mathbf{M}}\mathbf{M}\mathbf{F}\mathbf{n}^1\right|\right|_2^2,
\end{aligned}$$

where we used in the second step that $\mathbf{M}^2 = \mathbf{M}$, as $\mathbf{M}$ is a diagonal matrix with entries in $\{0, 1\}$. As we assumed the noise $\mathbf{n}^1$ to be zero-mean and independent of the noise $\mathbf{n}^0$, and the masks $\mathbf{M}$ and $\tilde{\mathbf{M}}$, it holds that

$$\mathop{\mathbb{E}}_{\mathbf{n}^1}\left[\Big\langle\tilde{\mathbf{M}}\mathbf{M}\mathbf{F}\big(\mathrm{f}_\theta(\tilde{\mathbf{v}}^0) - \mathbf{v}^*\big), \tilde{\mathbf{M}}\mathbf{M}\mathbf{F}\mathbf{n}^1\Big\rangle\right] = 0.$$

Therefore we get

$$\mathop{\mathbb{E}}_{\mathbf{n}^1}\left[\left|\left|\tilde{\mathbf{M}}\mathbf{M}\mathbf{F}\big(\mathrm{f}_\theta(\tilde{\mathbf{v}}^0) - \mathbf{v}^1_{xr}\big)\right|\right|_2^2\right] = \left|\left|\tilde{\mathbf{M}}\mathbf{M}\mathbf{F}\big(\mathrm{f}_\theta(\tilde{\mathbf{v}}^0) - \mathbf{v}^*\big)\right|\right|_2^2 + \mathop{\mathbb{E}}_{\mathbf{n}^1}\left[\left|\left|\tilde{\mathbf{M}}\mathbf{M}\mathbf{F}\mathbf{n}^1\right|\right|_2^2\right].$$

An analogous argument yields

$$\mathop{\mathbb{E}}_{\mathbf{n}^1}\left[\left|\left|\tilde{\mathbf{M}}^C\mathbf{M}\mathbf{F}\big(\mathrm{f}_\theta(\tilde{\mathbf{v}}^0) - \mathbf{v}^1_{xr}\big)\right|\right|_2^2\right] = \left|\left|\tilde{\mathbf{M}}^C\mathbf{M}\mathbf{F}\big(\mathrm{f}_\theta(\tilde{\mathbf{v}}^0) - \mathbf{v}^*\big)\right|\right|_2^2 + \mathop{\mathbb{E}}_{\mathbf{n}^1}\left[\left|\left|\tilde{\mathbf{M}}^C\mathbf{M}\mathbf{F}\mathbf{n}^1\right|\right|_2^2\right].$$

Using the fact that the joint masks $\tilde{\mathbf{M}}\mathbf{M}$ and $\tilde{\mathbf{M}}^C\mathbf{M}$ are orthogonal to each other and setting

$$c = \mathop{\mathbb{E}}_{\mathbf{M},\tilde{\mathbf{M}},\mathbf{n}^1}\left[\left|\left|(\tilde{\mathbf{M}}\mathbf{M} + 2\tilde{\mathbf{M}}^C\mathbf{M})\mathbf{F}\mathbf{n}^1\right|\right|_2^2\right],$$

yields the desired result.

Proof. [Proof of Proposition 1] For the sake of generality, we omit the assumption that the missing wedge masks $\mathbf{M}$ and $\tilde{\mathbf{M}}$ are non-overlapping and show that

$$\mathop{\mathbb{E}}_{\mathbf{M},\tilde{\mathbf{M}},\mathbf{n}^0,\mathbf{n}^1}\left[\left|\left|(\tilde{\mathbf{M}}\mathbf{M} + 2\tilde{\mathbf{M}}^C\mathbf{M})\mathbf{F}\Big(\mathrm{f}_\theta(\tilde{\mathbf{v}}^0) - \mathbf{v}^1_{xr}\Big)\right|\right|_2^2\right] = \mathop{\mathbb{E}}_{\mathbf{M},\tilde{\mathbf{M}},\mathbf{n}^0}\left[\left|\left|(\mathbf{I} - \tilde{\mathbf{M}}^C\mathbf{M}^C)\mathbf{F}\big(\mathrm{f}_\theta(\tilde{\mathbf{v}}^0) - \mathbf{v}^*\big)\right|\right|_2^2\right] + c, \quad (6)$$

where c is the constant from Lemma 1. Here, the mask $\mathbf{I} - \tilde{\mathbf{M}}^C \mathbf{M}^C$ zeros out all Fourier components that are contained in both missing wedges. If the wedges are non-overlapping, this joint mask reduces to the identity $\mathbf{I}$, and we obtain the result stated in Proposition 1.

By Lemma 1, in order to show Equation (6), it suffices to show that

$$\mathop{\mathbb{E}}_{\mathbf{M},\tilde{\mathbf{M}},\mathbf{n}^0}\left[\left\|(\tilde{\mathbf{M}}\mathbf{M} + 2\tilde{\mathbf{M}}^C\mathbf{M})\mathbf{F}\Big(\mathrm{f}_{\theta}(\tilde{\mathbf{v}}^0) - \mathbf{v}^*\Big)\right\|_2^2\right] = \mathop{\mathbb{E}}_{\mathbf{M},\tilde{\mathbf{M}},\mathbf{n}^0}\left[\left\|(\mathbf{I} - \tilde{\mathbf{M}}^C\mathbf{M}^C)\mathbf{F}\big(\mathrm{f}_{\theta}(\tilde{\mathbf{v}}^0) - \mathbf{v}^*\big)\right\|_2^2\right]. \quad (7)$$

Now we analyze the left-hand side of this equation. We fix the noise $\mathbf{n}^0$ and calculate

$$\begin{aligned}
&\mathop{\mathbb{E}}_{\mathbf{M},\tilde{\mathbf{M}}}\left[\left\|2\tilde{\mathbf{M}}^C\mathbf{M}\mathbf{F}\big(\mathrm{f}_{\theta}(\tilde{\mathbf{v}}^0) - \mathbf{v}^*\big)\right\|_2^2\right] \\
&= \mathop{\mathbb{E}}_{\mathbf{M},\tilde{\mathbf{M}}}\left[\left\|\tilde{\mathbf{M}}^C\mathbf{M}\mathbf{F}\big(\mathrm{f}_{\theta}(\tilde{\mathbf{v}}^0) - \mathbf{v}^*\big)\right\|_2^2\right] + \mathop{\mathbb{E}}_{\mathbf{M},\tilde{\mathbf{M}}}\left[\left\|\tilde{\mathbf{M}}^C\mathbf{M}\mathbf{F}\big(\mathrm{f}_{\theta}(\tilde{\mathbf{v}}^0) - \mathbf{v}^*\big)\right\|_2^2\right] \\
&= \mathop{\mathbb{E}}_{\mathbf{M},\tilde{\mathbf{M}}}\left[\left\|\tilde{\mathbf{M}}^C\mathbf{M}\mathbf{F}\big(\mathrm{f}_{\theta}(\tilde{\mathbf{v}}^0) - \mathbf{v}^*\big)\right\|_2^2\right] + \mathop{\mathbb{E}}_{\mathbf{M},\tilde{\mathbf{M}}}\left[\left\|\mathbf{M}^C\tilde{\mathbf{M}}\mathbf{F}\big(\mathrm{f}_{\theta}(\tilde{\mathbf{v}}^0) - \mathbf{v}^*\big)\right\|_2^2\right] \\
&= \mathop{\mathbb{E}}_{\mathbf{M},\tilde{\mathbf{M}}}\left[\left\|(\tilde{\mathbf{M}}^C\mathbf{M} + \mathbf{M}^C\tilde{\mathbf{M}})\mathbf{F}\big(\mathrm{f}_{\theta}(\tilde{\mathbf{v}}^0) - \mathbf{v}^*\big)\right\|_2^2\right].
\end{aligned}$$

For the second equation, we used that $\mathrm{P}(\mathbf{M}, \tilde{\mathbf{M}}) = \mathrm{P}(\tilde{\mathbf{M}}, \mathbf{M})$ and that the model input $\tilde{\mathbf{v}}^0$ depends on the masks $\mathbf{M}$ and $\tilde{\mathbf{M}}$ only through their product $\mathbf{M}\tilde{\mathbf{M}}$, for which the order of the masks does not play a role, thus the role of the two masks is exchangeable. For the last equation, we used that the masks $\tilde{\mathbf{M}}^C\mathbf{M}$ and $\mathbf{M}^C\tilde{\mathbf{M}}$ are orthogonal.

Using this equation and the fact that $\tilde{\mathbf{M}}\mathbf{M}$, $\tilde{\mathbf{M}}^C\mathbf{M}$ and $\mathbf{M}^C\tilde{\mathbf{M}}$ are orthogonal to each other, the left-hand side of Equation 7 becomes

$$\mathop{\mathbb{E}}_{\mathbf{M},\tilde{\mathbf{M}},\mathbf{n}^0}\left[\left\|(\tilde{\mathbf{M}}\mathbf{M} + \tilde{\mathbf{M}}^C\mathbf{M} + \mathbf{M}^C\tilde{\mathbf{M}})\mathbf{F}\big(\mathrm{f}_{\theta}(\tilde{\mathbf{v}}^0) - \mathbf{v}^*\big)\right\|_2^2\right] \overset{(*)}{=} \mathop{\mathbb{E}}_{\mathbf{M},\tilde{\mathbf{M}},\mathbf{n}^0}\left[\left\|(\mathbf{I} - \tilde{\mathbf{M}}^C\mathbf{M}^C)\mathbf{F}\big(\mathrm{f}_{\theta}(\tilde{\mathbf{v}}^0) - \mathbf{v}^*\big)\right\|_2^2\right],$$

where the equality $(*)$ holds because

$$\begin{aligned}
\tilde{\mathbf{M}}\mathbf{M} + \tilde{\mathbf{M}}^C\mathbf{M} + \mathbf{M}^C\tilde{\mathbf{M}} &= \tilde{\mathbf{M}}\mathbf{M} + (\mathbf{I} - \tilde{\mathbf{M}})\mathbf{M} + (\mathbf{I} - \mathbf{M})\tilde{\mathbf{M}} \\
&= \tilde{\mathbf{M}}\mathbf{M} + \mathbf{M} - \tilde{\mathbf{M}}\mathbf{M} + \tilde{\mathbf{M}} - \mathbf{M}\tilde{\mathbf{M}} \\
&= \mathbf{M} - \tilde{\mathbf{M}}\mathbf{M} + \tilde{\mathbf{M}} \\
&= \mathbf{I} - \tilde{\mathbf{M}}^C\mathbf{M}^C.
\end{aligned}$$

This concludes the proof of Equation 7 and Proposition 1.

4 IsoNet Reconstructions with Built-In Denoiser

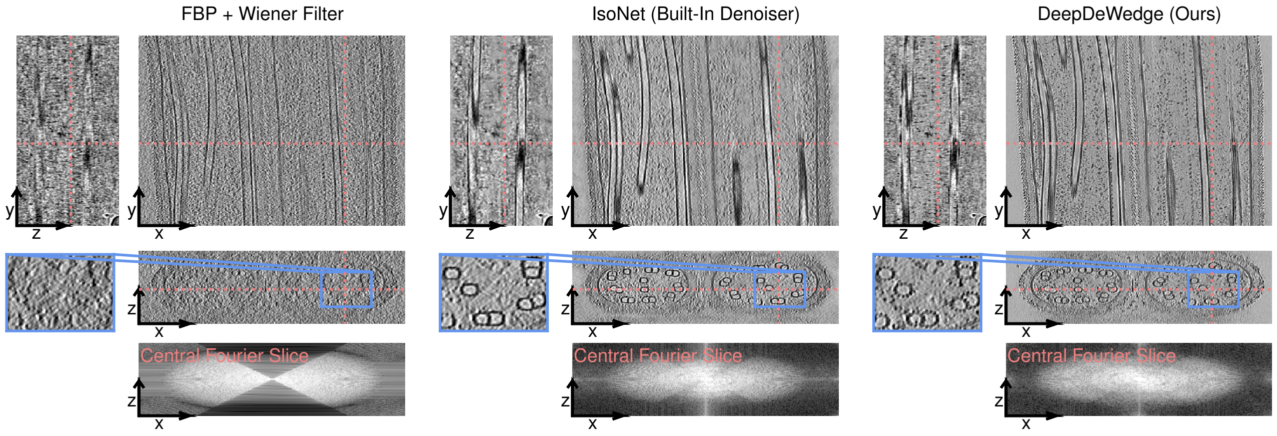

Supplementary Figure 1: Reconstructions of the *C. reinhardtii* flagella. IsoNet was fitted with its built-in Noisier2Noise-like denoisier rather than pre-denoising the FBP reconstruction with CryoCARE, resulting in a more noisy reconstruction.

5 Further Experiments

If not explicitly stated otherwise, we fitted all models on simulated tilt series from the first 3 tomograms of the SHREC 2021 Dataset with SNR 1/4. We performed only one run for each experiment. All remaining details regarding the model and optimizer are as in the Methods Section of the main document.

Applying DeepDeWedge to Multiple Tilt Series Simultaneously

As stated in the main body, and as done in some of our experiments, DeepDeWedge can be applied to multiple tilt series from similar samples simultaneously. Here, we discuss how this impacts performance compared to applying DeepDeWedge to each tilt series separately. We compare the performance of DeepDeWedge on the first 3 tomograms of the SHREC 2021 dataset for two scenarios:

- Collective fitting: We fitted DeepDeWedge for 1500 epochs on 150 sub-tomograms extracted from the FBP reconstructions of the three tilt series.
- Individual fitting: For each tilt series, we fitted one model for 3000 epochs on 50 sub-tomograms from the FBP reconstruction. We applied each model to the one tilt series used for its fitting.

We compared the best-case performances of collective fitting to individual fitting. Considering the average correlation coefficients with respect to the three ground truth tomograms, both approaches perform the same. However, all synthetic ground truth tomograms are very similar and densely packed with proteins. We expect collective fitting to be beneficial if the individual samples are similar but sparse, i.e. contain little signal of interest. For example, we found that collective fitting on all seven EMPIAR-10045 tilt series gave visually more appealing reconstructions than individual fitting. In case the samples are of different types, e.g. a combination of purified proteins or viruses and in-situ data, we recommend individual fitting.

Influence of Sub-Tomogram Size on Performance

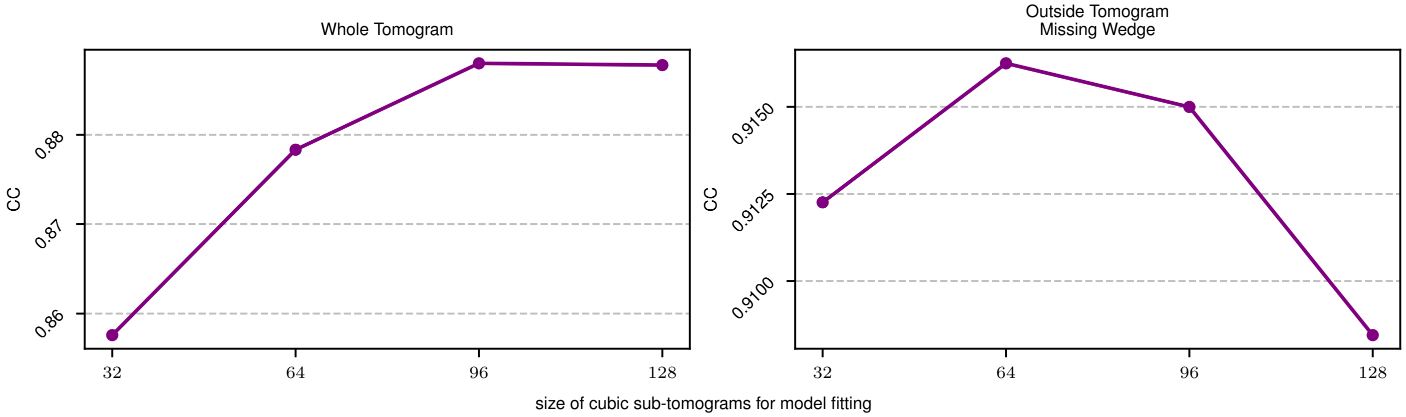

Supplementary Figure 2: Performance metrics for different sub-tomogram sizes used for model fitting.

The size of the sub-tomograms used for model fitting is a hyperparameter of DeepDeWedge, and here we investigate how it affects the performance. To this end, we repeated the experiment on synthetic data described for increasing the size of sub-tomograms. We scaled the total number of sub-tomograms for each run such that the number of voxels of the sub-tomogram fitting dataset is approximately constant. As for model fitting, we have to rotate the sub-tomograms, the sub-tomograms we extract have to be larger than the ones we actually use for fitting in order to avoid having to use padding. As a result, the maximum sub-tomogram size we could use for model fitting is $128 \times 128 \times 128$.

We fitted all models for 1000 epochs. During fitting, starting from epoch 500, we evaluated the models on all three tilt series every 100 epochs. We report the metrics for the epoch in which the highest correlation coefficient between reconstruction and ground-truth was achieved.

Figure 2 suggests that larger sub-tomograms for model fitting yield overall better performance than smaller ones. Note that the y-axis scales of the two plots are different and that the differences in the correlation coefficient outside the missing wedge (right plot) are very minor compared to the overall correlation coefficient (left plot).

6 Reconstructing Objects Perpendicular to the Electron Beam

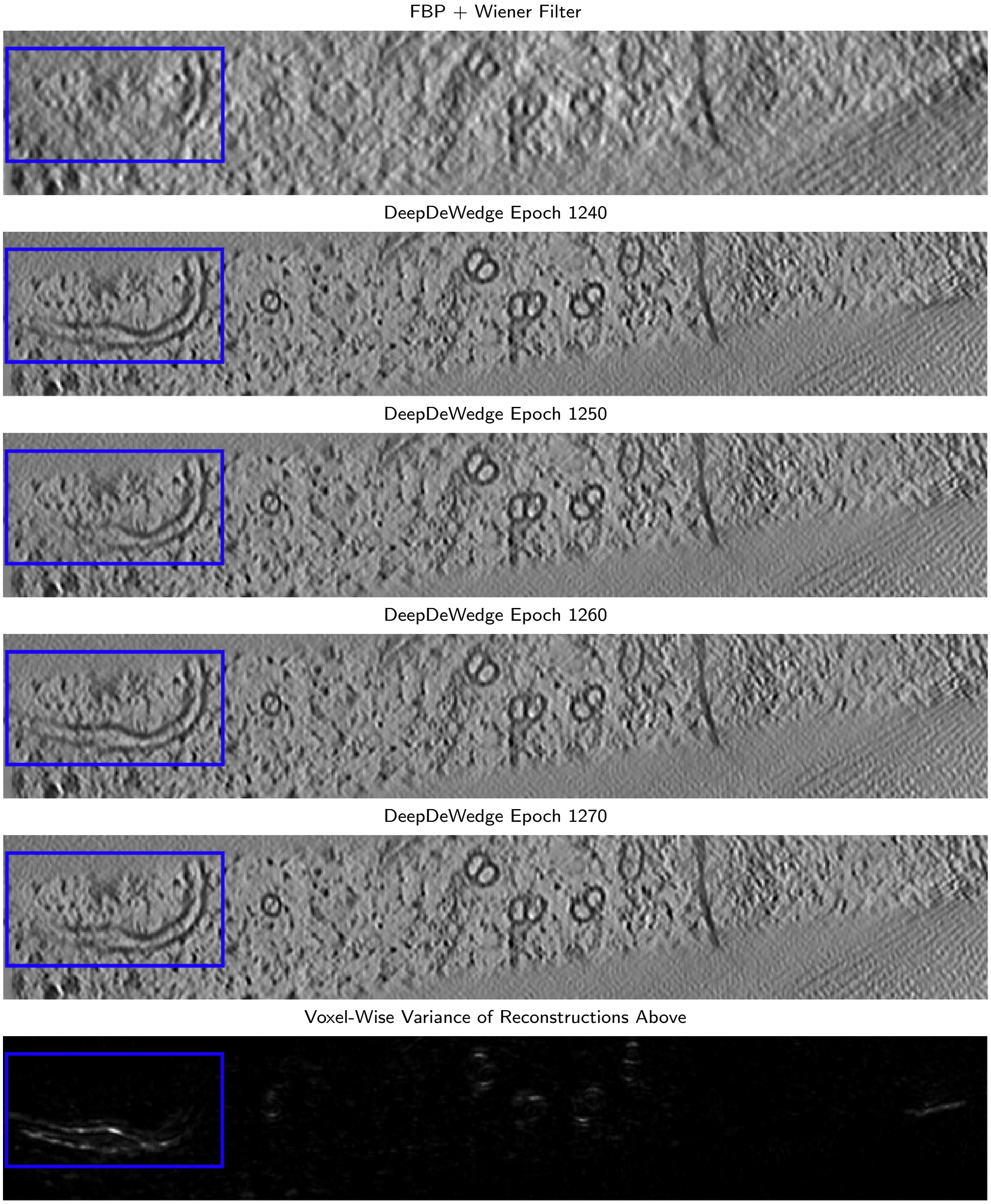

Supplementary Figure 3: Slices through reconstructions of Tomogram 3 from EMPIAR-11078.

Reconstructing objects perpendicular to the electron beam is difficult as many of their Fourier components lie inside the missing wedge region and are, therefore, not measured during tilt series acquisition. Nevertheless, we found that both DeepDeWedge and IsoNet can sometimes produce meaningful reconstructions of parts that are perpendicular to the electron beam, as can be seen, for example, in the x-z slice through the reconstructions of Tomogram 3 of EMPIAR-11078 shown in Figure 5.

Figure 3 displays an extreme case of a structure that is almost perfectly perpendicular to the electron beam. As the prediction in the blue box is based on very little data, and as there is no ground truth, it is unclear whether the predicted structure is correct. This is also indicated by the high voxel-wise variance over the course of different epochs in that area.

7 A Note on Hallucinations

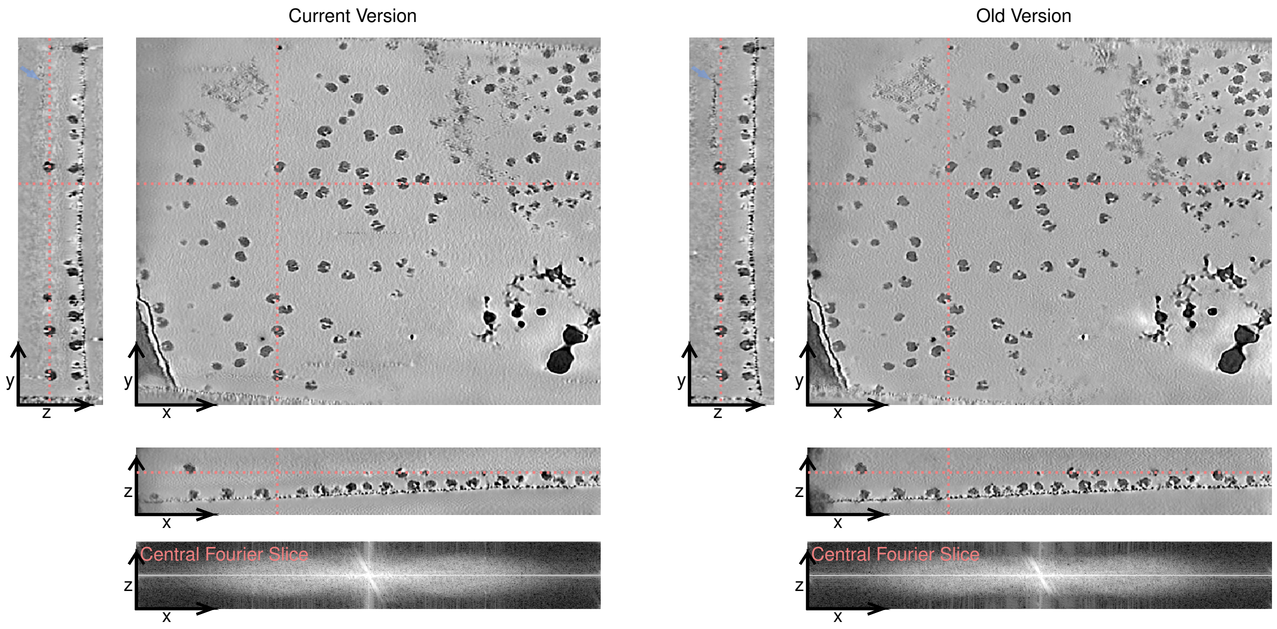

Supplementary Figure 4: Reconstructions of Tomogram 5 of EMPIAR-10045 obtained with the current version and an old version of DeepDeWedge.

We found that an earlier version of DeepDeWedge produces hallucinations or overpronounced details in the reconstructed tomograms. In Figure 4, we show the reconstruction of Tomogram 5 of EMPIAR 10045 obtained with this older version of DeepDeWedge, and the current version (left panel). We observe that with the old version, the reconstruction shows some unexpectedly strong densities at the air-water interface, which we marked with blue arrows. We do not observe such hallucinations in the volume reconstructed with the current version of DeepDeWedge.

We believe that the hallucinations shown in Figure 4 result from a mismatch between the model inputs used during model fitting and the inputs used for final reconstruction in the older version of the algorithm. In the older version of DeepDeWedge, the final refinement step, i.e. Step 3, was slightly different than the one presented in the Results section of the main paper: We used the FBP reconstruction of the full, non-splitted tilt series as input to the fitted model. Moreover, we did not progressively fill in the missing wedges of the model inputs during fitting in the previous version. This resulted in said mismatch: During fitting, the model received sub-tomograms from the FBP reconstructions of the splitted tilt series with an additional missing wedge as input. These are noisier and have stronger artifacts than the sub-tomograms of the full FBP reconstruction with only the original missing wedge. In the current version of DeepDeWedge, we try to minimize this mismatch by progressively filling the missing wedge of the model inputs during fitting and refining the FBP reconstructions of the splitted tilt series.

Finally, we note that we observed similar hallucinations as the ones shown in Figure 4 with our re-implementation of Cryo-CARE when applying the fitted model to the FBP reconstruction of the full tilt series.

Supplementary References

[1] Lehtinen, J. et al. Noise2Noise: learning image restoration without clean data. In: International Conference on Machine Learning (2018).

[2] Moran, N., Schmidt, D., Zhong, Y. & Coady, P. Noisier2noise: learning to denoise from unpaired noisy data. In: IEEE Conference on Computer Vision and Pattern Recognition (2020).

[3] Klug, T., Atik, D. & Heckel, R. Analyzing the sample complexity of self-supervised image reconstruction methods. In: Conference on Neural Information Processing Systems (2023).

[4] Millard, C. & Chiew, M. A theoretical framework for self-supervised mr image reconstruction using sub-sampling via variable density Noisier2Noise. IEEE Transactions on Computational Imaging **9**, 707–720 (2023).

[5] Millard, C. & Chiew, M. Simultaneous self-supervised reconstruction and denoising of sub-sampled MRI data with Noisier2Noise. Preprint at https://arxiv.org/abs/2210.01696 (2022).
