# Supplementary material for: A deep learning method for simultaneous denoising and missing wedge reconstruction in cryogenic electron tomography
Source: Nat Commun. 2024 Sep 23;15:8255. doi: 10.1038/s41467-024-51438-y (PMC11420219; doi:10.1038/s41467-024-51438-y)
Supplement: Supplementary file 3 — Reporting Summary [file 41467_2024_51438_MOESM3_ESM.pdf]

nature portfolio

Corresponding author(s): Reinhard Heckel

Last updated by author(s): 7/4/2024

Reporting Summary

Nature Portfolio wishes to improve the reproducibility of the work that we publish. This form provides structure for consistency and transparency in reporting. For further information on Nature Portfolio policies, see our Editorial Policies and the Editorial Policy Checklist.

Statistics

For all statistical analyses, confirm that the following items are present in the figure legend, table legend, main text, or Methods section.

n/a	Confirmed	
☒	☐	The exact sample size (*n*) for each experimental group/condition, given as a discrete number and unit of measurement
☒	☐	A statement on whether measurements were taken from distinct samples or whether the same sample was measured repeatedly
☒	☐	The statistical test(s) used AND whether they are one- or two-sided<br>*Only common tests should be described solely by name; describe more complex techniques in the Methods section.*
☒	☐	A description of all covariates tested
☒	☐	A description of any assumptions or corrections, such as tests of normality and adjustment for multiple comparisons
☒	☐	A full description of the statistical parameters including central tendency (e.g. means) or other basic estimates (e.g. regression coefficient) AND variation (e.g. standard deviation) or associated estimates of uncertainty (e.g. confidence intervals)
☒	☐	For null hypothesis testing, the test statistic (e.g. *F*, *t*, *r*) with confidence intervals, effect sizes, degrees of freedom and *P* value noted<br>*Give P values as exact values whenever suitable.*
☒	☐	For Bayesian analysis, information on the choice of priors and Markov chain Monte Carlo settings
☒	☐	For hierarchical and complex designs, identification of the appropriate level for tests and full reporting of outcomes
☒	☐	Estimates of effect sizes (e.g. Cohen's *d*, Pearson's *r*), indicating how they were calculated

Our web collection on statistics for biologists contains articles on many of the points above.

Software and code

Policy information about availability of computer code

Data collection	We did not collect data ourselves
Data analysis	IMOD 4.12.59 (for FBP in EMPIAR 11078 experiments)<br>IMOD 4.11.24 (for FBP in C. reinhardtii flagella experiments)<br>IsoNet 0.2.1<br>DeepDeWedge (no version number, method proposed in our paper, https://github.com/MLI-lab/DeepDeWedge)<br>Python 3.8.15<br>Python packages:<br>- tomosipo 0.6.0<br>- ts_algoritms (ts_algorithms @ git+https://github.com/ahendriksen/ts_algorithms.git@d9741ef53dbed2f9750e0e484f9af38b7f774051) (for FBP in EMPIAR 10045 and SHREC 2021 experiments)<br>- PyTorch Lightning 1.8.0.post1<br>- PyTorch 1.12.0 (with CUDA 11.3) (for neural network training)<br>- PyTorch 2.2.0 (with CUDA 11.8) (upgraded while writing this paper; does not influence results)<br>- Numpy 1.24.1<br>- Scipy 1.10.0

For manuscripts utilizing custom algorithms or software that are central to the research but not yet described in published literature, software must be made available to editors and reviewers. We strongly encourage code deposition in a community repository (e.g. GitHub). See the Nature Portfolio guidelines for submitting code & software for further information.

Data

Policy information about availability of data

All manuscripts must include a data availability statement. This statement should provide the following information, where applicable:

- Accession codes, unique identifiers, or web links for publicly available datasets
- A description of any restrictions on data availability
- For clinical datasets or third party data, please ensure that the statement adheres to our policy

All tomograms shown in Figure 3, Figure 4, Figure 5 and Figure 7 have been deposited to Figshare and are available at https://figshare.com/articles/journal_contribution/Tomograms_shown_in_Figures_of_the_DeepDeWedge_Paper/26169538.
The EMPIAR-10045 dataset used in this study is available in the EMPIAR database under accession code 10045 (https://www.ebi.ac.uk/empiar/EMPIAR-10045/).
The tilt series collected from the C. reinhardtii flagella is available at https://download.fht.org/jug/cryoCARE/Tomo110.zip.
The EMPIAR-11078 dataset used in this study is available in the EMPIAR database under accession code 11078 (https://www.ebi.ac.uk/empiar/EMPIAR-11078/).
The SHREC 2021 dataset is available at https://dataverse.nl/dataset.xhtml?persistentId=doi:10.34894/XRTJMA.

Research involving human participants, their data, or biological material

Policy information about studies with human participants or human data. See also policy information about sex, gender (identity/presentation), and sexual orientation and race, ethnicity and racism.

Reporting on sex and gender	N/A
Reporting on race, ethnicity, or other socially relevant groupings	N/A
Population characteristics	N/A
Recruitment	N/A
Ethics oversight	N/A

Note that full information on the approval of the study protocol must also be provided in the manuscript.

Field-specific reporting

Please select the one below that is the best fit for your research. If you are not sure, read the appropriate sections before making your selection.

☒ Life sciences ☐ Behavioural & social sciences ☐ Ecological, evolutionary & environmental sciences

For a reference copy of the document with all sections, see nature.com/documents/nr-reporting-summary-flat.pdf

Life sciences study design

All studies must disclose on these points even when the disclosure is negative.

Sample size	EMPIAR 10045: 7 tomograms<br>CryoCARE tutorial dataset: 1 tomogram<br>EMPIAR 11078: 3 tomograms<br>SHREC 2021 (synthetic data): 3 tomograms<br>We chose the number of tomograms based on either availability or based on the amount of data necessary to fit DeepDeWedge.<br>We did not perform statistical analysis on this data.
Data exclusions	From the seven EMPIAR 10045 tomograms, we only show Tomogram 5 which is also shown in the IsoNet paper. This selection criterion was pre-determined.<br>From the three EMPIAR 11078 tomograms we used for model fitting, we only show the first two. We ommited the third tomogram because its FBP reconstruction had artifacts, suggesting corrupt data or an issue in the reconstruction process.<br>From the three SHREC 2021 tomograms we used for model fitting, we only show the first one, as they all look very similar. This selection criterion was pre-determined.
Replication	Our results can be replicated by applying DeepDeWedge to the publicly available datasets used in our work.
Randomization	N/A as we did not perform any group allocation
Blinding	N/A as we did not perform any group allocation

Reporting for specific materials, systems and methods

We require information from authors about some types of materials, experimental systems and methods used in many studies. Here, indicate whether each material, system or method listed is relevant to your study. If you are not sure if a list item applies to your research, read the appropriate section before selecting a response.

Materials & experimental systems

n/a	Involved in the study
☒	☐ Antibodies
☒	☐ Eukaryotic cell lines
☒	☐ Palaeontology and archaeology
☒	☐ Animals and other organisms
☒	☐ Clinical data
☒	☐ Dual use research of concern
☒	☐ Plants

Methods

n/a	Involved in the study
☒	☐ ChIP-seq
☒	☐ Flow cytometry
☒	☐ MRI-based neuroimaging

Plants

Seed stocks	N/A
Novel plant genotypes	N/A
Authentication	N/A
